# Multi-modal digital twin model outperforms conventional biomarker stratification in pancreatic cancer

**DOI:** 10.3389/frai.2026.1873681

**Published:** 2026-08-25

**Authors:** Matthew Griffiths, Jennifer J. Knox, Grainne M. O’Kane, Trevor J. Pugh, Julie M. Wilson, Anna Dodd, Sandra E. Fischer, Robert C. Grant, Faiyaz Notta, Steven Gallinger, Irina Babina, Andrew V. Biankin

**Affiliations:** 1 Concr Limited, London, United Kingdom; 2 Wallace McCain Centre for Pancreatic Cancer, Princess Margaret Cancer Centre, University Health Network, Toronto, ON, Canada; 3 St. Vincent’s University Hospital and School of Medicine, University College Dublin, Dublin, Ireland; 4 Ontario Institute for Cancer Research, Toronto, ON, Canada; 5 Princess Margaret Cancer Centre, University Health Network, University of Toronto, Toronto, ON, Canada; 6 Department of Medical Biophysics, University of Toronto, Toronto, ON, Canada; 7 Department of Pathology, University Health Network, Toronto, ON, Canada; 8 Wolfson Wohl Cancer Research Centre, School of Cancer Sciences, College of Medical, Veterinary and Life Sciences, University of Glasgow, Glasgow, United Kingdom

**Keywords:** Bayesian, biomarker, digital twin, foundation model, pancreatic cancer, stratification

## Abstract

Better patient selection for treatment is critical to improving both cancer care and therapeutic development in oncology. The ability to predict individual patient responses to cancer treatment ahead of time would transform cancer care with substantial impact on outcomes, quality of life and cost. We generated molecular digital twins of individual participants using a Bayesian foundation model of cancer (FarrSight®) in the COMPASS clinical trial (a non-randomized study of mFOLFIRINOX and gemcitabine + nab-paclitaxel in first-line advanced pancreatic cancer). We compared these individual digital twin predictions to the existing Moffitt classification of pancreatic ductal adenocarcinoma. Individual digital twin predictions of response to mFOLFIRINOX outperformed the Moffitt classification in the basal-like subtype with an AUC of 72.3% compared to the conventional biomarker AUC of 44.8%; overall accuracy of 65.8% vs. 47.4%; PPV of 60% vs. 40%; and NPV of 72.2% vs. 52.2%. Individual patient response predictions using models such as FarrSight® have the potential to better select patients for treatment with established therapeutics and in therapeutic development compared to biomarkers based on population averages.

## Introduction

Pancreatic ductal adenocarcinoma (PDAC) remains one of the most lethal malignancies, with a 5-year survival of 13% (Siegel et al., 2023). The clinical management of PDAC is hindered by prognostic markers with only limited clinical utility (Sohal et al., 2020; Humphris et al., 2012), a lack of predictive biomarkers for current therapies, and few actionable molecular aberrations for existing drugs (Waddell et al., 2015). Transcriptomic profiling has established the Moffitt classification (Moffitt et al., 2015), which divides PDAC into “Classical” and “Basal-like” subtypes. The basal-like subtype is associated with poor outcome and represents the population most in need of better therapeutic stratification. However, no predictive tool has achieved routine clinical adoption. This gap is exacerbated in early-phase clinical trials, where small cohort sizes render conventional statistical biomarker methods underpowered, directly impeding trial success. Here we show that a Bayesian Digital Twin Model (FarrSight®) is capable of utilizing biological insights from large-scale pre-clinical and pan-cancer datasets to predict response in a relatively small cohort of patients.

## Methods

### Patient cohort

Participants in the COMPASS trial (NCT02750657) (Aung et al., 2018; Knox et al., 2025). The COMPASS trial was a non-randomized study of mFOLFIRINOX and gemcitabine + nab-paclitaxel in first-line advanced pancreatic cancer, where participants underwent molecular profiling.

**Figure 1:**
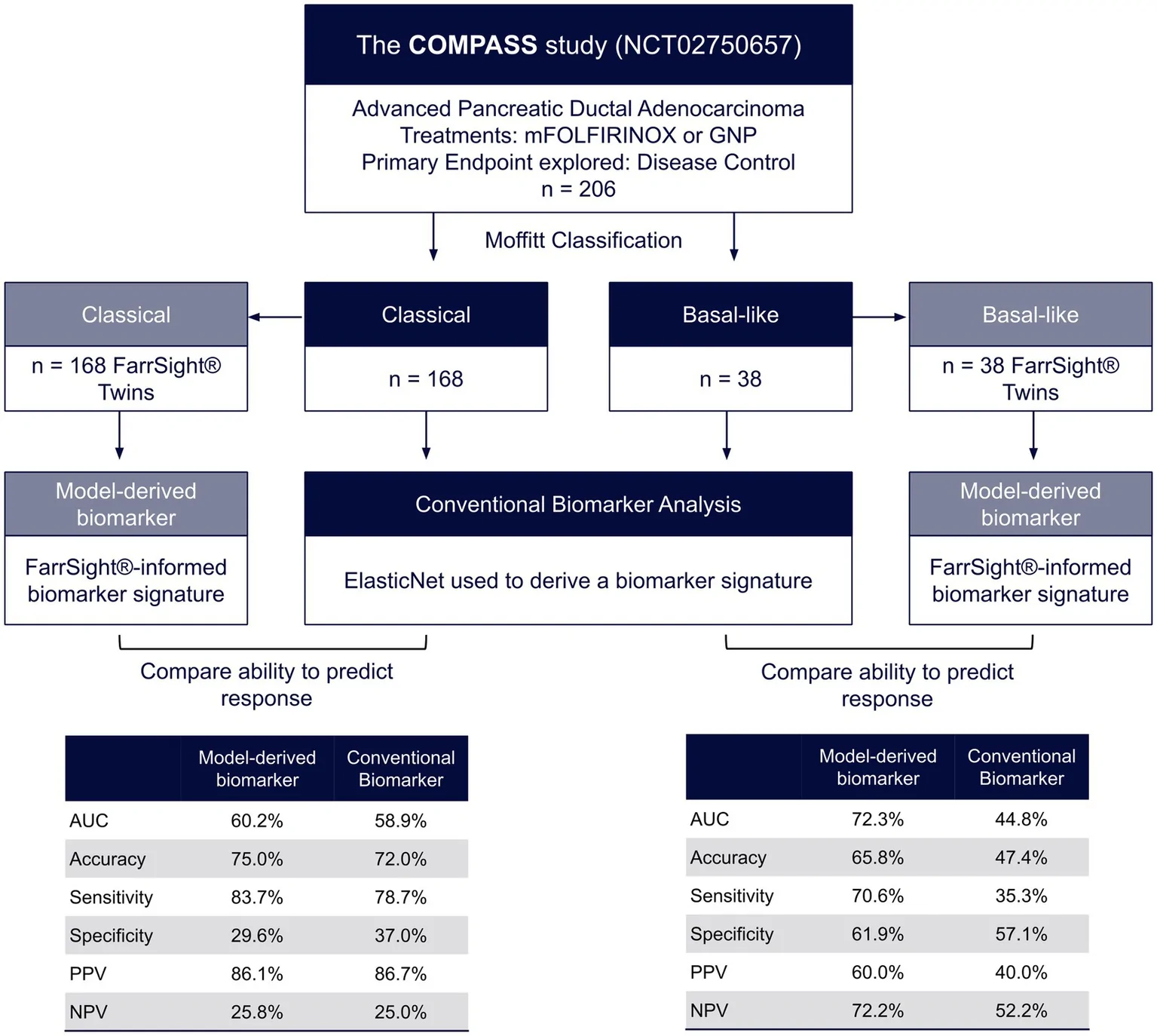
Overview of the analysis and accuracy results. AUC, Area under the receiver operating characteristic curve; PPV, Positive predictive value; NPV, Negative predictive value; mFOLFIRINOX, Leucovorin (folinic acid), 5-fluorouracil, irinotecan, and oxaliplatin; GNP, Gemcitabine and nab-paclitaxel.

Biomarker signature-generation: 1. The FarrSight® model is a composite Bayesian model trained on a multi-dimensional dataset of 25 billion data points, incorporating 190,005 patients with a variety of cancer types; 17,633 compounds; 890 cell lines; and 335 patient-derived xenograft (PDX) models [model architecture and method published previously (Griffiths et al., 2024)]. The model imputes missing data across modalities and utilizes transfer learning to bridge pre-clinical dose–response signals into clinical predictions. This trained model was used to generate feature importance data. Candidate genes in the FarrSight® analysis were ranked by relative importance at the individual patient level and aggregated to derive a response signature. To avoid overfitting the model for predictions of treatment response, the model was further trained using cross-fold splits of the cohort where the model was trained on four of the splits and predicted on the fifth omitted split, with five iterations. 2. Conventional Biomarker: A standard informatics approach where predictive gene signatures from transcriptomic gene expression data were selected using ElasticNet using a modified Moffitt classifier (Aung et al., 2018; Knox et al., 2025; Zou and Hastie, 2005; Breiman, 2001). The endpoints evaluated were Disease Control (DC), defined by RECIST criteria (DC was defined as stable disease (SD) or better vs. progressive disease (PD)), and overall survival. Figure 1 provides an overview of the analysis, including accuracy metrics.

### Biological feature analysis

In the Conventional Biomarker approach, the analysis was restricted to the top 50 most variably expressed genes across the training cohort. For the FarrSight® model, the top 50 most important gene expression features in the cross-fold training sets were used. No imputation of gene expression was performed in this step, and no other patient features were used.

## Results

### Predictive accuracy across sub-types

The COMPASS trial (NCT02750657) was a non-randomized study of participants with advanced PDAC who received first-line chemotherapy mFOLFIRINOX or gemcitabine + nab-paclitaxel. Comprehensive clinico-pathological and multi-omic data were available for 206 patients, including whole-genome sequencing, RNA-Seq with modified Moffitt classification, and therapeutic response data (Aung et al., 2018; Knox et al., 2025; Zou and Hastie, 2005; Breiman, 2001).

In the 168 Classical subtype patients, the FarrSight®-derived biomarker was comparable to the Moffitt classification across a range of accuracy metrics, with area under the curve (AUC) of 60.2% vs. 58.9%, respectively, and similar positive/negative predictive values for the two biomarkers (Figure 1, bottom left panel). Due to class imbalance and choice of threshold, some of the metrics are unbalanced for the classical subtype. The FarrSight® and conventional biomarkers significantly enriched Disease Control in the classical subtype patients [logOR = 1.0 (0.2, 1.8), Fisher’s exact *p* = 0.02] vs. [logOR = 1.1 (0.3, 1.9), Fisher’s exact *p* = 0.01]. When evaluating the AUC per split, there was no significant difference *p* = between the AUCs for the FarrSight® and conventional biomarkers [60.6%, (45.3, 75.9%) vs. 57.9%, (43.9, 71.9%)].

However, in the 38-patient basal-like subtype, FarrSight® demonstrated superior predictive accuracy compared to the conventional biomarker across all metrics. The FarrSight®-derived biomarker signature achieved an AUC of 72.3% compared to the conventional biomarker AUC of 44.8%; overall accuracy of 65.8% vs. 47.4%; PPV of 60% vs. 40%; and NPV of 72.2% vs. 52.2% (Figure 1, bottom right panel). The poorer performance of the conventional approach was likely driven by the model not having enough information to generalize due to small patient numbers and worse feature choices; it is likely that with larger patient numbers, the conventional performance would increase.

**Figure 2:**
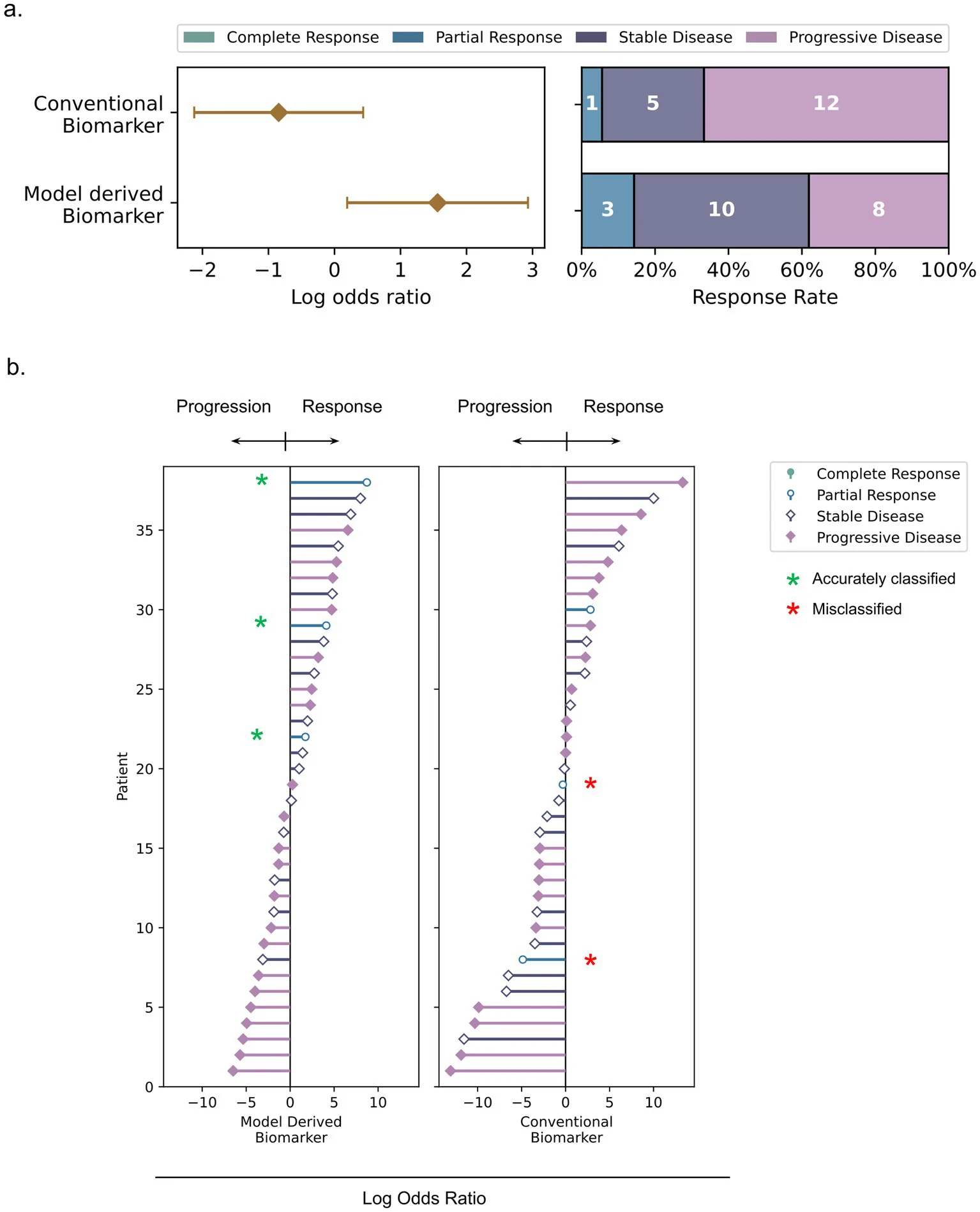
Model-derived biomarker outperformed conventional biomarker in basal-like group. **(A)** Stratification of disease control in Moffitt classification basal-like patients using a model-derived biomarker vs. a conventional elastic net biomarker identified by cross-validation. The conventional approach did not significantly enrich disease control (log odds ratio −0.9, 95% CI − 2.1 to 0.4; Fisher’s exact *p* = 0.95), whereas the model-derived biomarker significantly improved disease control (log odds ratio 1.6, 95% CI 0.2 to 2.9; Fisher’s exact *p* = 0.02). **(B)** Lollipop plot shows cross-validated predicted log odds ratio relative to the baseline response rate of disease control for both models across 38 basal-like patients. The model-derived biomarker correctly stratified all three partial responders, whereas the conventional model misclassified two.

As a consequence, we focused on the basal-like subtype and explored the performance of the two biomarker approaches in this group of 38 patients (17 cases of disease control and 21 cases of progressive disease). Stratifying patients based on the FarrSight®-derived biomarker captured a significantly higher rate of disease control in patients with the basal-like subtype, achieving a statistically robust enrichment [logOR = 1.6 (0.2, 2.9), Fisher’s exact *p* = 0.02]. In contrast, the conventional biomarker failed to stratify for response (log odds ratio −0.9, 95% CI − 2.1 to 0.4; Fisher’s exact *p* = 0.95; Figure 2a). There was a significant difference in enrichment (log OR 2.4, 95% CI 0.5 to 4.3, *p* = 0.005), though the difference between the cross-fold AUCs for the FarrSight® and conventional biomarkers [72.4%, (47.6, 97.3%) vs. 48.3%, (24.1 to 72.4%)] was not significant, likely due to the limitation evaluating AUC in the small cross-folds (Airola et al., 2011).

**Figure 3:**
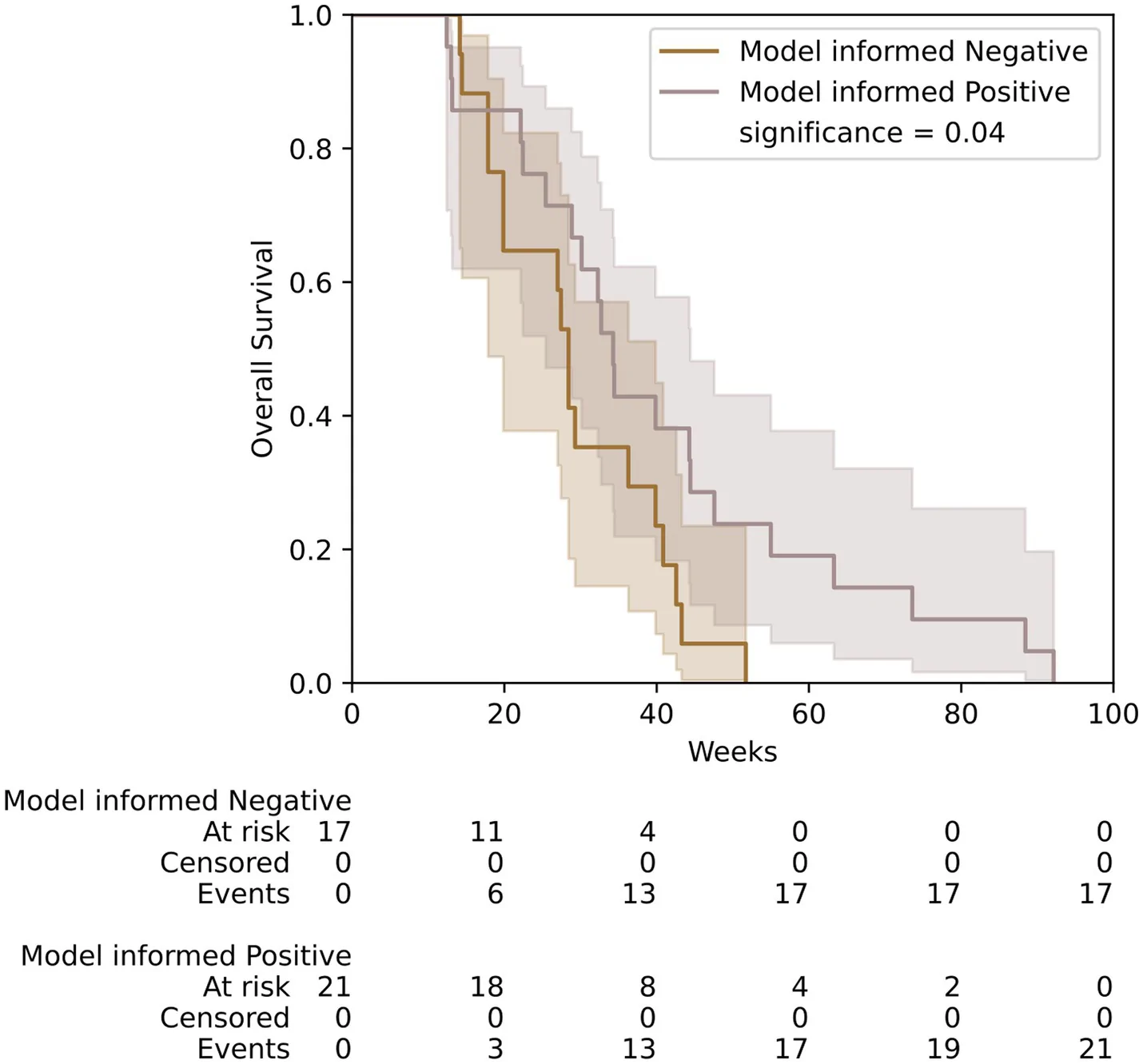
Digital twin predictions show a significant difference in survival. Kaplan–Meier curves compare overall survival between biomarker-enriched (positive) and non-enriched (negative) groups. Survival was significantly improved in the enriched group (log-rank *p* = 0.04). The biomarker was trained using disease control data, while feature importance incorporated contributions from both response and survival predictions.

Predicting individual patient response ahead of treatment would enhance clinical decision-making, particularly for those who will not respond to mFOLFIRINOX, avoiding unnecessary toxicity and the potential to explore other therapeutic options. At an individual patient level, FarrSight® accurately classified all three partial responders within the basal-like cohort, whereas the standard biomarker approach misclassified 2 of these (Figure 2b). These are small numbers and further studies would be required to validate the significance. Nonetheless, the FarrSight®-derived stratification translated into a significant difference in survival (Figure 3).

### Biological feature importance

**Figure 4:**
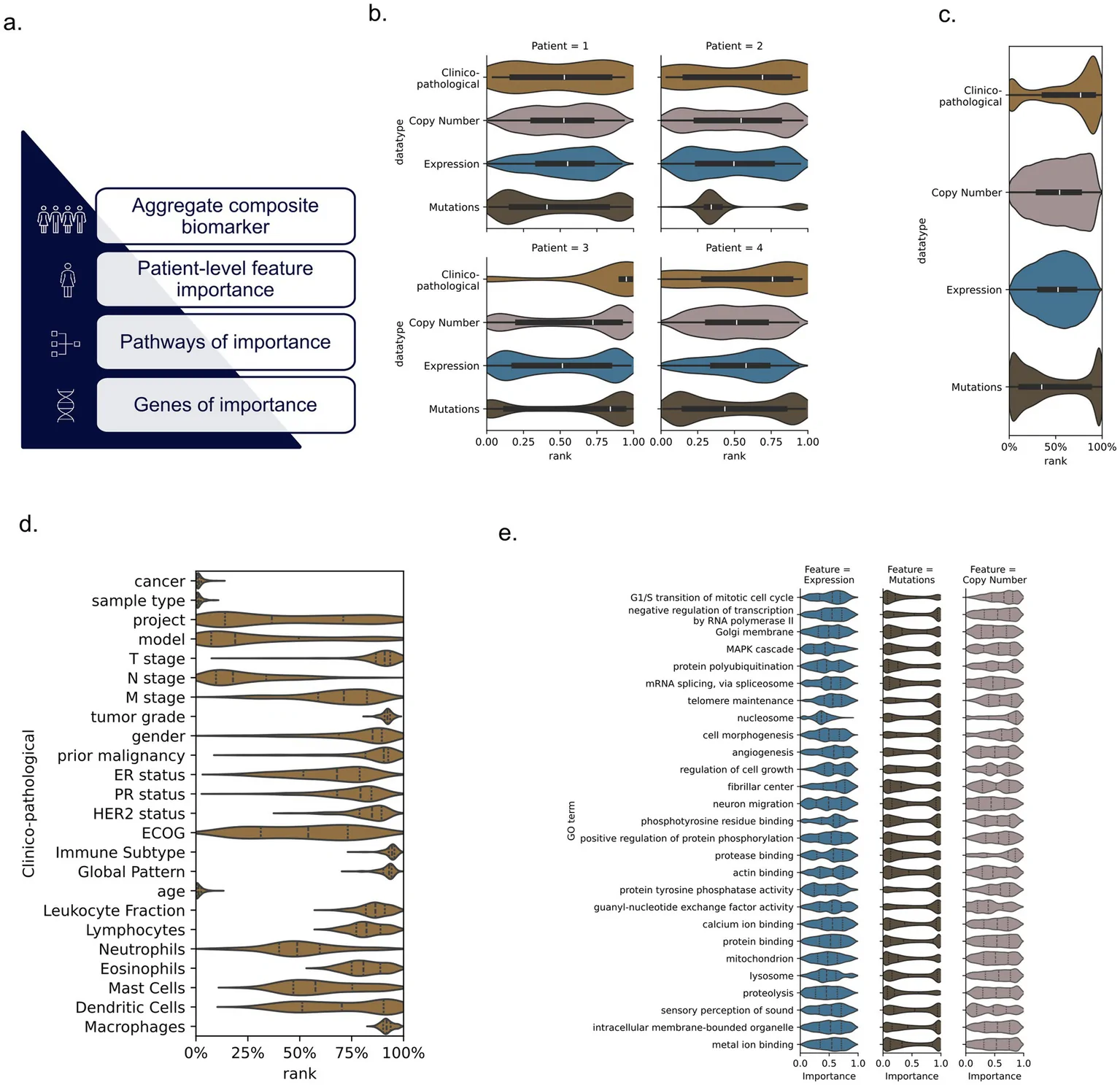
Model-derived biomarker is based on feature importance. **(a)** Distribution of patient-specific feature importance scores, shown as violin plots of percentile ranks across all features. **(b)** Examples illustrate inter-patient variability in feature importance profiles. **(c)** Aggregate distribution of feature importance across all patients in the COMPASS cohort. **(d)** Distribution of feature importance for the clinicopathological features. **(e)** Genomic feature importance stratified by data type (expression, mutation, copy number) and grouped by associated Gene Ontology (GO) terms, highlighting pathway-level contributions.

Unlike conventional models that apply fixed feature weightings to the overall cohort, FarrSight® defines the importance of specific features at the level of an individual patient that can infer potential biological mechanisms of response and resistance and then aggregate into cohorts (Figure 4a). Analysis of feature importance revealed significant patient-to-patient variability, with four examples depicted in Figure 4b. In some patients, clinico-pathological features dominated the prediction (patient 3), while in others, gene expression or mutational profiles carried greater weight. The variability in importance demonstrates that the model is able to identify specific mechanisms associated with response and resistance in individual patients, with the potential for more tailored approaches to treatment choice. Aggregating these features for a cohort-level analysis, clinico-pathological features had the highest median importance, while copy number variation and expression exhibited the most even spread (Figure 4c). Mutations were dichotomous (present or not), either predicted to be highly important or not important between individuals. Within clinico-pathological features, cancer type, sample type, and age were considered the least important in predicting therapeutic response to chemotherapy (Figure 4d). Tumor grade, immune infiltrate, and global pattern (of immune infiltrate) were considered the most important. These features were derived from the immune analysis performed by Thorsson et al. (2018) and Bailey et al. (2016).

Finally, we explored molecular features across mutation, CNV and Gene Expression FarrSight® ranked pathways considered to be drivers of chemotherapy response by the model. While biological analysis of the signaling pathways is outside the scope of this report, we compared how the pathways identified approach compared to the reported biological hallmarks of the basal-like subtype (Figure 4e).

Biological mechanisms detected by FarrSight® that have been previously reported Bailey et al. (2016), Rhim et al. (2012), Eser et al. (2013), Witkiewicz et al. (2015), and Vennin et al. (2018).

*RNA and alternative splicing*: Alternative splicing (specifically driven by factors such as QKI) is a master regulator that promotes the plastic, undifferentiated state of basal-like tumors. FarrSight® independently identified “mRNA splicing, via spliceosome” as a highly ranked, consistent driver of chemotherapy response prediction across multiple data modalities. *Epigenetic and transcriptional reprogramming*: The basal-like state is maintained by global epigenetic shifts (e.g., chromatin remodeling, histone acetylation) that suppress classical epithelial genes. FarrSight® highly ranked GO terms directly related to this, including “negative regulation of transcription by RNA polymerase II” and “nucleosome”. *Kinase networks and phosphorylation*: Basal-like tumors rely heavily on altered kinase signaling (such as FAK and ErbB) to drive aggressive growth and anoikis resistance (survival without attachment). FarrSight® heavily prioritized features such as “MAPK cascade,” “positive regulation of protein phosphorylation,” “phosphotyrosine residue binding,” and “protein tyrosine phosphatase activity”.” Cell Cycle Control: Extreme cell cycle deregulation is a hallmark of this highly proliferative, aggressive subtype. FarrSight® identified “G1/S transition of mitotic cell cycle” as one of the most prominent cross-modal features the model used to predict response (Rhim et al., 2012). *Angiogenesis and cellular structure*: Basal-like tumors are characteristically hypoxic and undergo Epithelial-to-Mesenchymal Transition (EMT), changing their cellular structure to metastasize. FarrSight® flagged “angiogenesis,” “cell morphogenesis,” and “actin binding” as key predictive indicators.

### Unique to FarrSight® (identified by the model, less prominent in standard basal subtyping)

*Organelle-specific dynamics*: FarrSight® placed surprisingly high importance on specific cellular machinery, including the “Golgi membrane,” “lysosome,” and “mitochondrion.” While metabolic shifts are known in PDAC, the model’s pinpointing of these specific organelles for drug response prediction highlights its ability to find granular, actionable vulnerabilities. *Telomere maintenance*: Ranked highly across multiple data types by the FarrSight®. While telomere shortening is an early event in general PDAC development, the model found it to be a specific, robust differentiator between responders and non-responders to standard chemotherapy in this advanced basal-like cohort. *Sensory/extracellular signaling*: The model flagged unusual GO terms such as “sensory perception of sound,” which likely points to the co-option of specific mechano-transduction or atypical receptor signaling pathways in the tumor microenvironment.

### Unique to existing literature (known biology, not explicitly highlighted by FarrSight®)

*Specific axon guidance pathways*: The SEMA3A-NRP1 axis is heavily documented in recent literature as a basal-like driver, but did not appear in the top functional GO terms shown. *Specific immune niche signaling*: While the FarrSight® does use clinical features such as “Immune Subtype,” “Macrophages,” and “Lymphocytes” to build its predictions, the specific molecular cytokines (such as CXCL10/CXCR3 networks) driving the basal-like immune microenvironment were not listed in the top genomic feature outputs.

## Concluding remarks

This application of the FarrSight® Digital Twin model to the COMPASS trial demonstrates the potential of digital twins to outperform conventional, static biomarker models in stratifying the highly aggressive basal-like subtype of PDAC. This approach aligns with the paradigm shift of moving beyond rigid “one biomarker for one drug” strategies toward multi-modal *in silico* simulations of individual biological trajectories. By leveraging large pan-cancer Bayesian prior probabilities to impute missing modalities, FarrSight® attempts to address the persistent challenge of incomplete and inconsistent multi-omics data in precision medicine.

These preliminary findings must be interpreted with caution as proof of concept; small cohorts are highly susceptible to selection bias, and the reliance on data imputation introduces statistical uncertainty that may not fully capture the profound spatial and temporal heterogeneity of PDAC. Nonetheless, this early evidence demonstrates that such Bayesian frameworks hold significant promise for optimizing shared decision-making for patients with severe malignancies.

## Data Availability

The raw data supporting the conclusions of this article will be made available by the authors, without undue reservation. The COMPASS data is available via EGA.
