# Supplementary material for: Multi-modal digital twin model outperforms conventional biomarker stratification in pancreatic cancer
Source: Front Artif Intell. 2026 Aug 25;9:1873681. doi: 10.3389/frai.2026.1873681 (PMC13550003; doi:10.3389/frai.2026.1873681)
Supplement: Supplementary file 1 [file Supplementary_File_1.docx]

Supplementary Methods

**Supplementary Fig 1. Summary of data used to train the FarrSight® model.**

Summary Upset plot of data used to train FarrSight® model. Totals, CCLE=845, GENIE=168,913, ICGC=10,268, PDXE=281, TCGA=10,922.

The FarrSight® model was designed to simultaneously solve multiple tasks:

- Learn interactions between features and treatments
- Missing feature imputation (cancer type, stage, RNAseq, mutations, copy-number)
- Generalise across multiple systems (HTS, cell line, PDX, patient)
- Predict multiple outputs (Gene targets, dose viability, IC50, Response, Survival)

All the outputs of the model are Bayesian distributions to quantify model predictive uncertainty. In this case the core feature used was the learn interactions between the molecular features, and treatment that drive the predicted outcome.

The model was trained independently on this dataset (see Supp Fig 1.) and was applied naively to the COMPASS dataset, any imputation performed on the data was naive to/untrained on the dataset. The feature importances of treatment-specific response were generated by the model for each patient, and these were used to drive biomarker discovery. The feature importance calculation also used other information about the patients (when available) such as mutation, copy number variation, and clinical features such as age, sex, TNM staging. The Feature importances used to generate these analyses are available on request through our online platform for reproducibility.

The cross-fold splits were generated with a 5-fold split with random seed = 42, stratified by the reported RECIST response (See Supp Table 1). Both the Standard and FarrSight® biomarkers were trained and tested on identical splits. Feature selection was performed on each training cross-fold separately to avoid data leakage. The biomarkers were generated using a scikit-learn LogisticModel with an elasticnet penalty, and hyperparameters, solver='saga', C=1e1, l1_ratio=0.5, max_iter=5000. These models were trained on only the measured RNAseq expression, no imputation was used to generate the features for the models

| **Moffitt Subtype** | **Cross Fold** | **Progressive Disease** | **Disease Control** | | |
| --- | --- | --- | --- | --- | --- |
|  |  |  | **Stable Disease** | **Partial Response** | **Complete Response** |
| Basal-like | 0 | 5 | 2 | 1 | 0 |
|  | 1 | 4 | 3 | 1 | 0 |
|  | 2 | 4 | 3 | 1 | 0 |
|  | 3 | 4 | 3 | 0 | 0 |
|  | 4 | 4 | 3 | 0 | 0 |
| Classical | 0 | 6 | 16 | 12 | 0 |
|  | 1 | 6 | 16 | 12 | 0 |
|  | 2 | 5 | 15 | 13 | 1 |
|  | 3 | 5 | 15 | 13 | 0 |
|  | 4 | 5 | 15 | 13 | 0 |
| Supp Table 1. Breakdown of responses in training cross-folds. This shows that each training set contained at least 16 PD and 12 Disease Control participants. | | | | | |

In this analysis Disease Control (RECIST CR, PR and SD, compared to progressive disease) was chosen as the primary endpoint as it provided the best numbers for comparison as PR is very uncommon in the Basal-like subtype of PDAC. It is also well accepted that due to the extensive desmoplastic stroma characteristic of PDAC, RECIST criteria for response are imperfect as the fibrosis often does not change in size with epithelial malignant cell response. When evaluating the accuracy of the predictions the threshold for disease control was chosen to be 50%.
